# Autophagy regulation and viral exploitation: insights into African swine fever virus pathogenesis

**DOI:** 10.3389/fcimb.2026.1733035

**Published:** 2026-02-16

**Authors:** Ran Li, Xuyan Liu, Yuetong Lei, Jinjing Bao, Zhaolin Zhang, Xiaoyong Chen

**Affiliations:** 1 Xingzhi College, Zhejiang Normal University, Jinhua, China; 2 Zhejiang Combiwell Health Products Technology Development Co. Ltd., Jinhua, China

**Keywords:** African swine fever virus, autophagy, pathogenesis, viral replication, virus-host interaction

## Abstract

African swine fever virus (ASFV), a devastating pathogen of swine, poses a great threat to the global pork industry and food supply due to its high lethality and lack of effective countermeasures. In this review, we aim to elucidate the intricate interplay between ASFV and host autophagy-a cellular process with dual roles in viral pathogenesis. By dissecting the molecular mechanisms through which ASFV interacts with autophagy, this review resolves key controversies surrounding autophagy’s context-dependent effects on viral replication, immune evasion, and tissue damage. The significance of this work lies in its potential to bridge current knowledge gaps by unraveling how autophagy-modulating viral proteins dictate divergent outcomes in infection, identifying novel therapeutic targets to counteract ASFV immune evasion strategies, and providing a framework for understanding host-pathogen conflicts in large DNA virus infections. Overall, we hope that proposing autophagy-targeted interventions could act as a paradigm shift in developing urgently needed antiviral strategies against ASFV.

## Introduction

African swine fever (ASF), caused by the African swine fever virus (ASFV), is a highly contagious hemorrhagic disease of pigs with resulting in substantial lethality rates exceeding 90% among susceptible animals (Blome et al., 2020). First identified in Kenya in 1921, ASF remained endemic in sub-Saharan Africa for decades, sustained by a sylvatic cycle involving wild suids and soft ticks of the *Ornithodoros* genus (Gaudreault et al., 2020). The first reported outbreak of ASF in Europe occurred in Portugal in 1957, after which the virus gradually spread across much of Western Europe over the following three decades (Cwynar et al., 2019). Notably, its introduction to Georgia in 2007 marked the beginning of a Eurasian epidemic, and expanded further to central and eastern Europe, culminating in severe outbreaks in China and across Southeast Asia, devastating pork industries and destabilizing global markets (Galindo and Alonso, 2017; Rock, 2021; Bourry et al., 2022). This virus exhibits complex transmission dynamics, including direct pig-to-pig contact, fomites, tick vectors, wild boar, and aerosols, enabling rapid spread in both free-range and intensive farming systems (Guinat et al., 2016; Main et al., 2022a, b). During quiescent periods, subclinical infections persist, as demonstrated by detection of ASFV in asymptomatic slaughter pigs, suggesting silent viral circulation and economic underreporting. In China, the 2018–2019 epidemic led to the culling of the national swine herd, causing an estimated $140 billion in direct losses and triggering a surge in global pork prices (Li et al., 2022b). Similarly, in sub-Saharan Africa, recurring outbreaks disrupt rural economies, where pigs serve as critical assets for poverty alleviation (Njau et al., 2021). Globally, ASF socioeconomic impact extends beyond direct livestock losses, affecting feed industries, labor markets, and food security. Its persistence in wild reservoirs and capacity for asymptomatic carriage in other animals underscore the intractable challenge of eradication (Quembo et al., 2018; Williams et al., 2024). Notably, no WHO- or OIE-approved vaccine are available as of 2026. While live attenuated vaccines show promise, safety concerns, such as residual virulence and viral shedding, hinder approval (Wang et al., 2024b). Subunit and vector-based vaccines targeting proteins like P72 or P54 often fail to confer sterilizing immunity due to weak neutralizing antibody responses (Petrovan et al., 2020; Miao et al., 2023). Although newer platforms (nanocarrier-adjuvanted and mRNA vaccines) offer improved protection, their efficacy against diverse ASFV strains is inconsistent, and reactogenicity remains an issue (Song et al., 2023; Gong et al., 2024). High costs, cold-chain requirements, and fragmented regulation further limit accessibility, underscoring the need for integrated control strategies combining vaccination and robust biosecurity.

**Figure 1:**
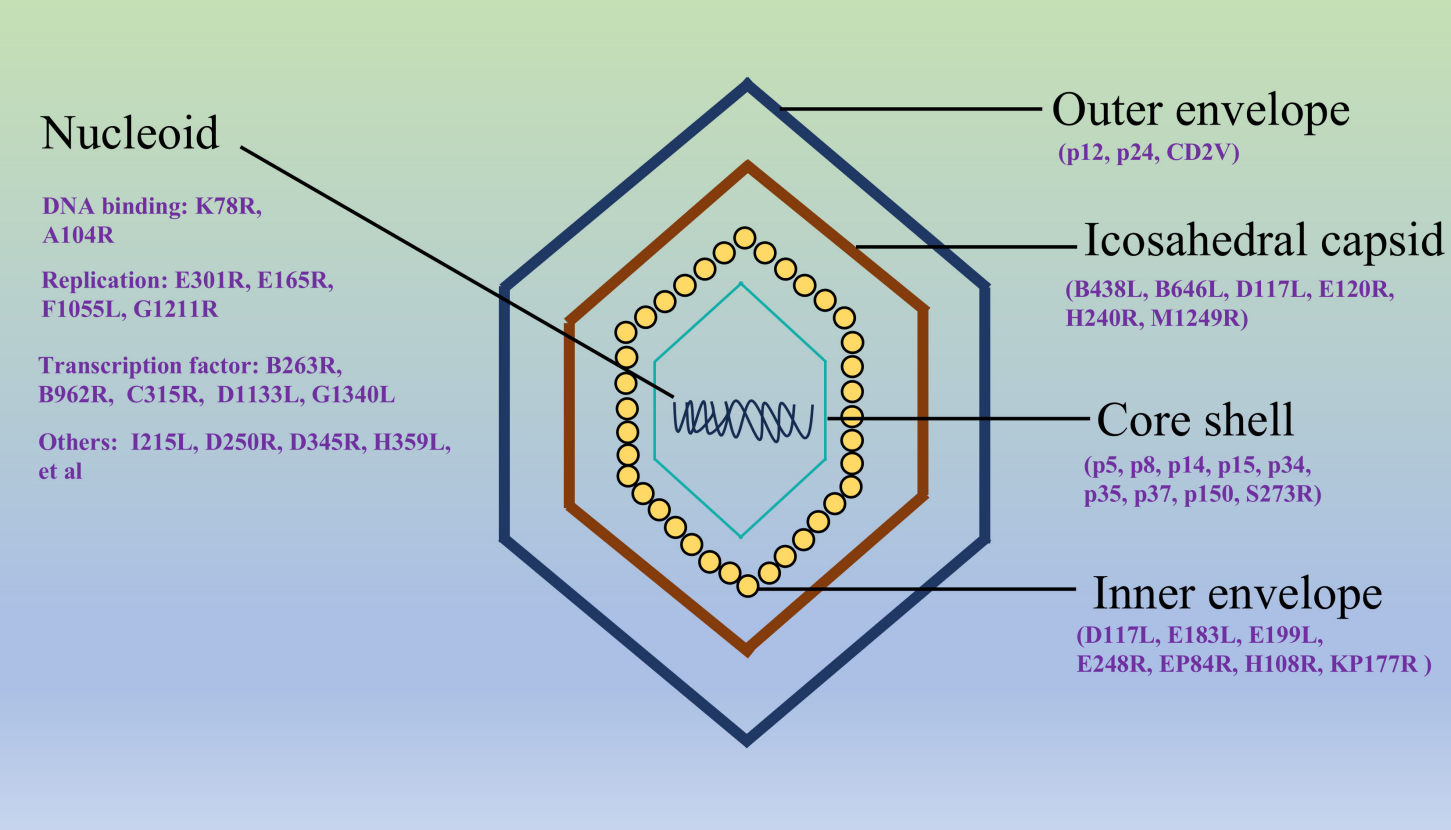
The structure of the ASFV virion. The outermost lipid envelope is acquired through budding from the host cell’s plasma membrane. Beneath this envelope lies the icosahedral capsid (250 nm maximum diameter), which assembles on the cytoplasmic side of the viral inner membrane. Concentric to the capsid, the core shell (180 nm maximum diameter) forms a protective layer surrounding the central nucleoid. This nucleoid complex contains the viral genome (a double-stranded DNA) along with associated proteins essential for genome packaging and early viral replication processes. The structural arrangement demonstrates the viral sophisticated architecture for protecting its genetic material while facilitating host infection (Dixon, 2025).

ASFV, the sole member of the *Asfarviridae* family and *Asfivirus* genus, is a large, enveloped, double-stranded DNA virus (**Figure 1**), responsible for a highly contagious and lethal hemorrhagic disease in domestic and wild swine (Ruedas-Torres et al., 2024). Its genome, ranging from 170 to 190 kilobases, is notable for its complexity and high variability, featuring terminal inverted repeats and tandem repeats that contribute to genetic diversity (Andrés, 2017). To date, 24 ASFV genotypes have been documented, among which genotype I and II recombinant strains pose the greatest threat to the pig farming industry (Quembo et al., 2018; Zhao et al., 2023).The ASFV genome encodes more than 150 open reading frames (ORFs), with approximately half involved in viral replication, structural assembly, and immune evasion (Hooper et al., 2024). Unlike most DNA viruses, ASFV replicates predominantly in the cytoplasm of infected cells, utilizing virally encoded enzymes for transcription and genome replication, a feature linked to its evolutionary adaptation to arthropod vectors (Dunn et al., 2020). ASFV virions exhibit a multilayered architecture: an inner nucleoprotein core (containing the genome and DNA-binding proteins), surrounded by a proteinaceous core shell, an inner lipid membrane enriched with viral transmembrane proteins, and an icosahedral capsid composed of the major capsid protein p72 (Dixon et al., 2013). The outermost envelope, derived from host membranes during budding, incorporates viral glycoproteins critical for host cell attachment and entry (Ren et al., 2022). ASFV encodes diverse virulence and immunomodulatory proteins. Structural proteins such as p72 (capsid), p54 (inner membrane), and p30 (early antigen) are essential for viral entry, assembly, and immune recognition (Neilan et al., 2004). Nonstructural proteins, including multigene family (MGF) proteins, antagonize host innate immunity by inhibiting interferon (IFN) responses and apoptosis (Gao et al., 2021). Enzymes like the DNA polymerase (PolX) and topoisomerase II facilitate viral genome replication and repair (Coelho and Leitão, 2020; Pérez-Núñez et al., 2024). Notably, ASFV’s ability to encode proteins that mimic host functions, such as A238L that strengthens innate immune via TANK-binding kinase 1 (TBK1)- interferon regulatory factor 3 (IRF3) axis, underscores its evolutionary sophistication in manipulating cellular signaling pathways (Liu et al., 2024). The viral genetic plasticity, combined with its intricate particle structure and diverse protein repertoire, enables robust host immune evasion and environmental persistence, posing significant challenges for vaccine development and disease control.

Autophagy is a conserved intracellular degradation process that maintains cellular homeostasis by recycling damaged organelles, protein aggregates, and pathogens via lysosomal digestion (Chen et al., 2024). It involves the formation of double-membraned autophagosomes that engulf cytoplasmic cargo, which subsequently fuse with lysosomes for enzymatic breakdown (Wang et al., 2021b). Key regulators, such as the mammalian target of rapamycin (mTOR)-UNC-51-like kinases 1(ULK1) axis, which orchestrate autophagosome biogenesis and cargo recognition (Liu et al., 2023). In viral infections, autophagy exhibits dual roles. On one hand, it acts as an innate antiviral defense mechanism, selectively targeting viral components for degradation (Viret et al., 2018). For instance, autophagy restricts replication of herpesviruses and influenza A virus (IAV) by degrading virions or viral proteins. Additionally, autophagy enhances antigen presentation to activate adaptive immunity and modulates inflammatory responses by suppressing excessive cytokine production (D’Arcy, 2019). Conversely, many viruses exploit or subvert autophagy to promote replication (Viret et al., 2018). Some pathogens inhibit autophagosome-lysosome fusion, creating stabilized autophagosomes as platforms for viral assembly. Others, like poliovirus and dengue virus, induce autophagy to provide membrane resources for replication complexes (Heaton and Randall, 2011; Aponte-Diaz et al., 2025). Hepatitis C virus (HCV) and SARS-CoV-2 co-opt autophagy-related proteins to facilitate virion maturation and egress (Chu and Ou, 2021; Ivanova et al., 2023). Furthermore, autophagy can suppress apoptosis, prolonging host cell survival to sustain viral production (Das et al., 2021). Understanding these dynamics offers therapeutic potential may enhance antiviral responses or disrupt viral exploitation. However, the dual nature of autophagy necessitates precise targeting to avoid exacerbating pathogenesis.

The precise mechanisms governing the interplay between ASFV and host autophagy machinery remain incompletely resolved, yet emerging evidence underscores the virus adept co-option of this pathway to amplify replication efficiency (**Table 1**). Intriguingly, certain host factors may conversely harness autophagy to restrict viral propagation, revealing a bidirectional tug-of-war at the host-pathogen interface (Yang et al., 2024; Song et al., 2025). Therapeutic strategies could involve disrupting viral co-option of autophagy or reprogramming the pathway to enhance antiviral defenses. However, the Janus-faced nature of autophagy-capable of both curbing and exacerbating infection-mandates context-specific modulation to avoid unintended consequences, such as amplifying inflammatory pathology or impairing cellular homeostasis. By dissecting the molecular logic of ASFV-autophagy crosstalk, future research must prioritize high-resolution mechanistic studies and *in vivo* validation of candidate targets. We hope that this review would not only refine fundamental understanding of viral pathogenesis but also catalyze the development of precision therapies, bridging the critical gap between basic virology and actionable solutions for ASF containment.

**Table 1:** ASFV proteins related to autophagy.

Name	Function	Viral replication
K205R	Activating ER stress and PERK-eIF2α cascade and promoting autophagosome formation	Negative (Wang et al., 2022b)
E199L	Suppressing PYCR2 expression and activating autophagy	Unknown (Chen et al., 2021)
EP153R	Modulating LAMP1/2 locations and blocking autophagosome-lysosome fusion	Unknown (Bai et al., 2024)
MGF505-7R	Mediating the autophagic degradation of STING by upregulating ULK1 and IRF7 to antagonize innate immunity	Positive (Li et al., 2021a)
L83L	Mediating autophagic degradation of STING	Positive (Cheng et al., 2023)
MGF505-6R	Facilitating the degradation of STING by autophagy	Positive (Yao et al., 2024)
MGF360-11L	Targeting TBK1 and IRF7 partly by autophagy	Positive (Yang et al., 2022a)
A137R	Promoting autophagic degradation of TBK1	Positive (Sun et al., 2022)
I215L	Promoting IRF9 degradation by autophagy	Positive (Li et al., 2022a)
MGF300-4L	Trigging autophagic degradation of IKKβ	Positive (Wang et al., 2024a)
MGF300-2R	Interacting with TOLLIP and promoting autophagic degradation of IKKα and IKKβ	Positive (Wang et al., 2023)
H240R	Interacting with and degrading NEMO to suppress the activation of NF-κB by autophagy	Positive (Zhou et al., 2022)
MGF360-4L	Targeting MDA5 for autophagic degradation by recruiting SQSTM1/p62	Positive (Sun et al., 2025)
MGF110-9L	Mediating the degradation of TBK1 by autophagy factor PIK3C2B	Positive (Zhu et al., 2023)
MGF505-7R	Mediating the degradation of TBK1 by PIK3C2B	Positive (Zhu et al., 2023)
P72	Delivered to autophagosome and degraded via interacting with STUB1 and SQSTM1/p62	Negative (Song et al., 2025)
MGF505-2R	Undergoing autophagic degradation by FoxJ1	Negative (Ma et al., 2022)
E165R	Degraded by FoxJ1 by autophagy	Negative (Ma et al., 2022)
CP204L	Degraded via SNX32-RAB1B-mediated autophagy	Negative (Yang et al., 2024)

## ASFV modulates autophagy in host cells via various pathways

Recent studies reveal that ASFV regulates autophagy through multiple interconnected pathways to support viral replication and immune evasion. One key mechanism involves the suppression of the mTORC1 signaling axis, a central regulator of autophagy initiation. ASFV protein K205R, expressed in early infection, directly interacts with the endoplasmic reticulum (ER) stress response, inducing unfolded protein responses that activate the PKR-like endoplasmic reticulum kinase (PERK)- eukaryotic initiation factor 2-alpha (eIF2α) cascade, thereby promoting autophagosome formation (Wang et al., 2022b). Additionally, ASFV E199L, a protein required for viral core entry, induces complete autophagy in cells by interacting with and downregulating pyrroline-5-carboxylate reductase 2 (PYCR2), a reductase critical for proline biosynthesis. Mechanistically, E199L-mediated suppression of PYCR2 expression activates autophagy, aligning with prior evidence linking PYCR2 depletion to autophagic induction (Chen et al., 2021). Intriguingly, another study uncovers a novel mechanism by which ASFV disrupts the autophagy-lysosome axis, a critical host defense pathway, to facilitate its replication. ASFV recruits lysosomal membrane proteins (LAMP1/2) to viral factories while suppressing autophagic flux in macrophages. Viral protein EP153R, a transmembrane glycoprotein, was identified as a key player that redistributes LAMP1/2 to the ER, blocking autophagosome-lysosome fusion. Although EP153R contributes to this process, an ASFV mutant lacking EP153R retained the ability to sequester LAMP1/2 into VFs and inhibit autophagy, suggesting functional redundancy by other viral proteins (Bai et al., 2024). Similarly, Shimmon et al. found that ASFV infection blocked the formation of autophagosomes by interacting with Akt and mTOR complex 2 (mTORC2), thereby suppressing autophagy (Shimmon et al., 2021). These multifaceted interactions highlight the dual role of ASFV-induced autophagy: while initially serving as a host defense mechanism, it is ultimately hijacked to create a pro-viral niche by balancing cell survival, nutrient recycling, and immune modulation.

## ASFV exploits autophagy to suppress innate immune response

**Figure 2:**
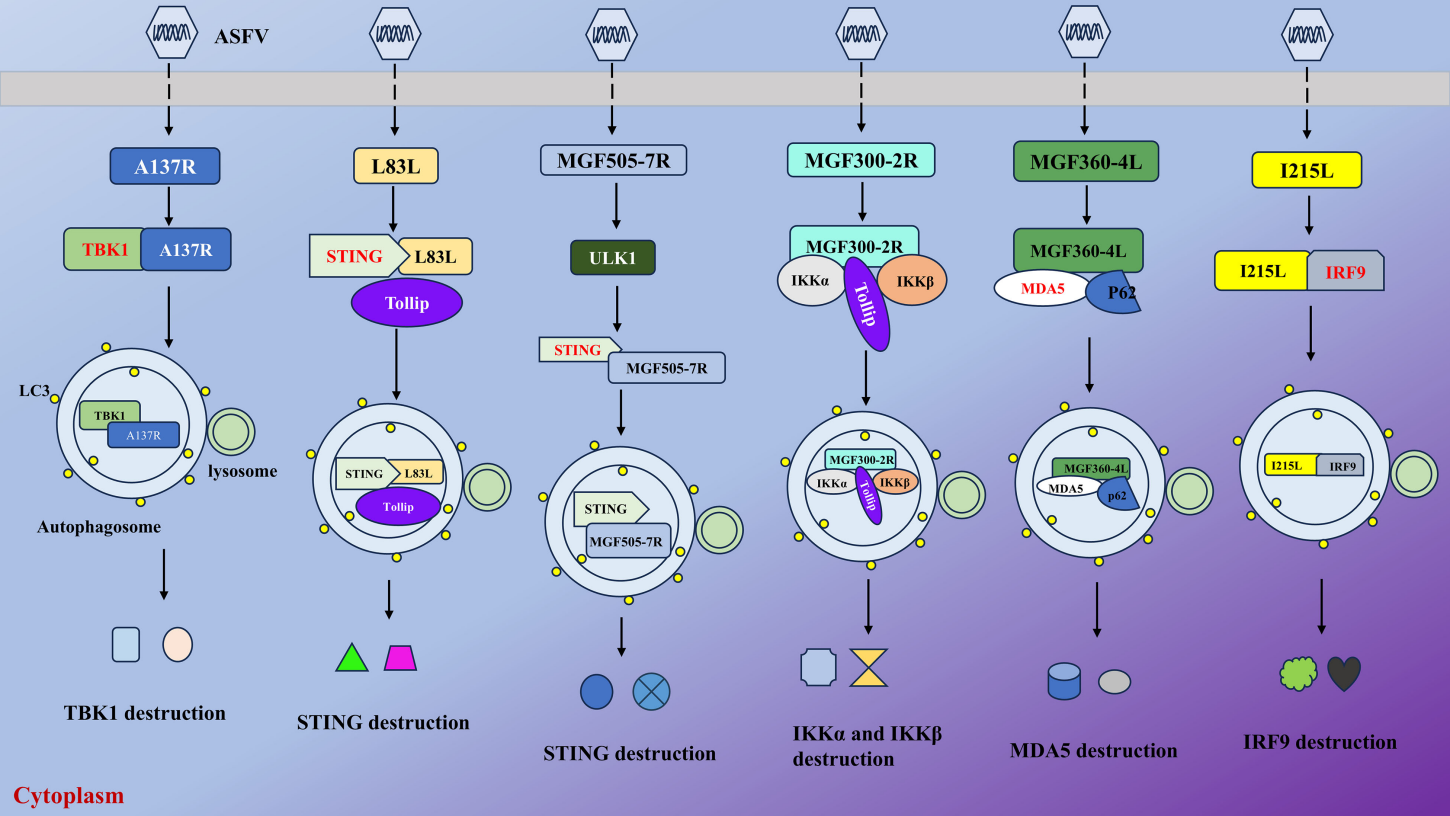
ASFV suppresses cellular innate immunity by targeting and degrading key factors to promote its infection. As illustrated, ASFV could exploit its-encoded proteins to target various factors, such as MDA5, TBK1, and STING. Then, they together with viral proteins and receptors (TOLLIP and SQSTM1/p62) are delivered to autophagosome for degradation, thus inhibiting the production of downstream antiviral agents.

Host innate immunity serves as the first line of defense against intruding pathogens and subsequently contributes to adaptive immunity (Chen et al., 2022). The cyclic GMP-AMP synthase (cGAS)-stimulator of interferon genes (STING) signaling axis provides a pivotal mechanism in host antiviral defense, initiating IFN-I production upon detection of cytosolic DNA from invading pathogens. STING is a critical innate immune protein that activates TBK1 and IFN responses to combat infections (Chen et al., 2016). As a large DNA virus, ASFV has evolved sophisticated immune evasion strategies by encoding multiple virulence factors that directly target the innate signaling to suppress IFN-I production (**Figure 2**). Mechanistically, these viral proteins disrupt critical nodes of the pathway-including cGAS enzymatic activity, STING oligomerization, and downstream TBK1-IRF3 phosphorylation-effectively blunting host immune surveillance. This coordinated suppression of DNA sensing pathways enables ASFV to establish persistent infection while evading innate immune clearance.

Li et al. showed that ASFV MGF505-7R, a member from the multigene family that could strengthen viral virulence and pathogenesis, directly interacts with STING, triggering its autophagic degradation via upregulation of the autophagy initiator ULK1. This degradation was evidenced by reduced STING levels in wild-type ASFV-infected cells and restored STING expression in MGF505-7R-deficient ASFV infections. Functional analyses demonstrated that MGF505-7R deletion substantially enhances IFN-β production and attenuated viral replication both *in vitro* and *in vivo* (Li et al., 2021a). Yang et al. discovered that MGF505-7R degrades IRF7 by autophagy to inhibit the production of IFN-I and ISGs (Yang et al., 2022b). Other proteins, L83L, a nonessential protein, associated with early viral replication, and MGF505-6R (also a member of the multigene family 505) also possess the same ability to facilitate autophagy-lysosomal degradation of STING, thus antagonizing cGAS-STING signaling (Cheng et al., 2023; Yao et al., 2024). Another study revealed that ASFV MGF360-11L targets TBK1, which is a serine/threonine protein kinase that functions in innate immune responses by phosphorylating downstream effectors like NF-κB, and IRF7 for degradation by autophagic pathway, thus inhibiting cGAS-mediated signaling (Yang et al., 2022a). Similarly, A137R, a structural protein affecting virulence, interacts with TBK1 and promotes its autophagic lysosomal degradation. This degradation disrupted the nuclear translocation of IRF3, thereby attenuating type I IFN production (Sun et al., 2022).

Additionally, Li et al. found that viral I215L, a nonstructural protein encoding a putative E2 ubiquitin conjugating enzyme, facilitates IRF9 for autophagic degradation to suppress IFN-I signaling (Li et al., 2022a). Recent studies showed that MGF300-4L, a member from MGF300 family, triggers the autophagic degradation of IKKβ and MGF300-2R recruited toll interacting protein (TOLLIP), a selective autophagy receptor that regulates cellular homeostasis by mediating the autophagic degradation of specific proteins and modulating immune signaling pathways, to destroy I kappa B kinase alpha (IKKα) and IKKβ to promote viral replication and pathogenicity (Wang et al., 2023, 2024a). The same group also elucidated a novel mechanism that viral capsid protein H240R suppresses activation of NF-κB by interacting with and degrading the nuclear factor-kappa B essential modulator (NEMO) via the autophagic pathway, thus reducing interleukin (IL)-1b transcription and inhibiting innate immunity (Zhou et al., 2022). In 2025, MGF360-4L, a member from the MGF360 family, was discovered to target melanoma differentiation-associated protein 5 (MDA5) by recruiting sequestosome 1 (SQSTM1)/p62 for autophagic degradation, thus suppressing IFN production and antagonizing innate immunity (Sun et al., 2025). Interestingly, Zhu et al. showed that deletions of MGF110-9L from MGF110 family and MGF505-7R from MGF505 family genes simultaneously facilitate IFN production by suppressing the degradation of TBK1 via an autophagy factor phosphatidylinositol 3-kinase (PI3K)-C2 beta (PIK3C2B) (Zhu et al., 2023).

## Host factors restrict ASFV replication via degradation of viral proteins

Viral proteins are essential for the virion assembly and replication. It has been shown that numerous viral proteins could be targeted and degraded via autophagy-lysosomal pathway, thus inhibiting viral replication and infection (Chen et al., 2023). For example, porcine reproductive and respiratory syndrome virus (PRRSV) infection could be suppressed by proteasome subunit b type 1 (PSMB1), which targets NSP12 for autophagic degradation (Chen et al., 2024). Porcine epidemic diarrhea virus (PEDV), a swine coronavirus, could interact with a multitude of host factors. These factors target various PEDV proteins, such as nucleocapsid (N) protein, S2 protein, and M protein, leading to the impaired viral replication (Wang et al., 2021a; Cao et al., 2025). These studies suggest that autophagy could be exploited by host to destroy viral proteins to inhibit virus replication.

ASFV P72 is a major capsid protein that undergoes ubiquitination by the E3 ubiquitin ligase STUB1, which subsequently is recognized by SQSTM1 and delivered to autophagosome for degradation. The STUB1-SQSTM1 axis facilitates P72 degradation and leads to impaired ASFV replication (Song et al., 2025). Forkhead Box J1 (FoxJ1), a modulator in immune cell development, has been shown to play an important role in suppressing ASFV replication. Mechanistically, it promotes the autophagic degradation of MGF505-2R from the MGF505 family and the dUTPase E165R proteins and in turn the cysteine protease S273R reduces the expression of FoxJ1 to weaken its effect (Ma et al., 2022). Moreover, sorting nexin 32 (SNX32), an important role in sorting and membrane trafficking of endosomal cargoes, has been indicated as a host factor that restricted ASFV replication. SNX32 was identified as a host protein interacting with CP204L, a multifunctional protein of ASFV. Silencing SNX32 enhances ASFV replication, while its overexpression suppresses it. Mechanistically, SNX32 recruits autophagy-related Ras-related protein 1B (RAB1B) to degrade CP204L, inhibiting viral growth. These findings reveal SNX32-RAB1B-mediated autophagy as a host defense mechanism against ASFV, offering insights for antiviral strategies (Yang et al., 2024).

## Perspectives and conclusion

The intricate interplay between autophagy regulation and viral pathogenesis, as exemplified by ASFV, underscores a dynamic battlefield where host defense mechanisms and viral countermeasures collide. ASFV’s exploitation of autophagy to degrade key immune signaling molecules reveals a convergent evolutionary tactic to cripple the cGAS-STING-TBK1 axis, a central hub for IFN-I production (Lin et al., 2024). These findings position autophagy not merely as a catabolic pathway but as a critical regulatory node in immune evasion. The selective degradation of STING and TBK1 highlights a hierarchical targeting strategy, wherein ASFV disrupts both cytosolic DNA sensing and downstream signaling, ensuring comprehensive suppression of IFN-I responses. Such mechanisms likely contribute to ASFV’s notorious resilience in host cells and its capacity to establish persistent infections.

Notably, selective autophagy receptors (SARs), such as SQSTIM1/p62, NDP52, and OPTN, function as critical mediators that recognize ubiquitinated viral components or pathogen-associated molecular patterns, thereby targeting them for lysosomal degradation. They have been shown to restrict RNA virus infections through multiple mechanisms: they can degrade viral nonstructural proteins or directly bind and deliver viral double-stranded RNA (dsRNA)-a potent pathogen-associated molecular pattern-to autophagosomes for destruction (Luo et al., 2024). Recent work reveals that the ER-resident protein disulfide isomerase PDIA3, a component linked to ER-phagy and the unfolded protein response, significantly suppresses EBOV replication by targeting cysteine residues in GP via its reductase activity, thereby reducing GP stability and virion incorporation (Wang et al., 2022a). Additionally, The ER-resident E3 ligase RNF185 mediates K27-linked polyubiquitination of GP1,2 at lysine 673, enabling its recognition by the autophagy receptor SQSTM1/p62 and subsequent ATG3/ATG5-dependent sequestration into autophagosomes. Thus, EBOV co-opts all three ER proteostasis arms-the calnexin cycle, ERAD, and reticulophagy-to downregulate GP1,2 via ubiquitin-directed lysosomal degradation, enhancing viral fitness and revealing unexpected crosstalk among these pathways (Zhang et al., 2022). These highlight how autophagy-related ER quality control pathways intersect with viral glycoprotein biogenesis-a vulnerability, underscoring that while selective autophagy serves as a potent barrier against diverse RNA viruses, successful pathogens have evolved sophisticated countermeasures that either dismantle SAR functionality or repurpose autophagic machinery for their benefit-principles highly relevant to understanding emerging viral threats such as ASFV, which, despite being a DNA virus, similarly manipulates autophagy to dampen innate immune signaling.

From a therapeutic perspective, the identification of autophagy-dependent immune evasion pathways opens new avenues for antiviral intervention. Pharmacological modulation of autophagy, either by inhibiting pathogenic autophagy flux hijacked by ASFV or by enhancing xenophagic clearance, could restore host defense mechanisms. However, the pleiotropic roles of autophagy in cellular homeostasis demand precision in targeting viral-specific interactions. For instance, small molecules blocking the binding of MGF505-7R to STING or A137R to TBK1, or suppressing the autophagic factors, like TOLLIP, STUB1 or SQSTM1/p62, could preserve IFN-I signaling without globally disrupting autophagy. However, the clinical translation of autophagy modulators in viral infections faces significant challenges, including the dual role of autophagy as both an antiviral defense and a pro-viral mechanism exploited by diverse pathogens, which complicates therapeutic timing, dosing, and context-specific efficacy. Moreover, the lack of virus- or pathway-selective autophagy regulators raises concerns about off-target effects on essential cellular homeostasis, potentially exacerbating immunopathology or impairing host resilience. Similarly, engineered live-attenuated vaccines with deletions in MGF505-7R or A137R represent promising candidates, as these mutants exhibit attenuated virulence while eliciting robust IFN-driven immunity, as demonstrated in recent studies (Li et al., 2021b; Koltsov et al., 2024).

Despite these ongoing advances, critical questions remain unresolved. First, the full repertoire of ASFV proteins that interface with autophagy machinery is yet to be cataloged. Systematic screens for viral interactors of ATGs or regulators may uncover additional immune evasion nodes. Second, the spatiotemporal regulation of autophagy during ASFV infection-whether autophagosome formation, lysosomal fusion, or substrate selectivity is preferentially manipulated-warrants deeper investigation. Third, the interplay between autophagy and other cell death pathways in ASFV pathogenesis remains poorly understood. Given that ASFV infection can both induce and suppress apoptosis depending on the infection stage, autophagy may serve as a rheostat balancing pro-survival and pro-death signals to optimize viral spread.

In conclusion, the study of ASFV pathogenesis through the lens of autophagy regulation has not only unraveled key mechanisms of viral immune evasion but also redefined autophagy as a double-edged sword in host-pathogen interactions. These insights bridge fundamental virology and translational immunology, offering a roadmap for rational vaccine design and host-directed therapies. Future research must prioritize *in vivo* validation of autophagy-targeted interventions and explore cross-species conservation of these mechanisms, which could inform broader antiviral strategies against DNA viruses exploiting similar tactics. By decoding the “autophagy paradox” in ASFV infection, we move to tipping the balance in favor of host defense, ultimately curbing the devastating impact of this pathogen on global agriculture.
